# Microbiome Profiling and Antimicrobial Efficacy of a Novel Silver‐Nanoparticle Hydrogel Dressing Against Clinical Isolates From Paediatric Burn Wounds

**DOI:** 10.1002/mbo3.70383

**Published:** 2026-08-24

**Authors:** Anteneh Amsalu, Anton Alvaro, Linda Quinn, Anna Antipov, Sylvia Sapula, Henrietta Venter, Bernard Carney, Hanif Haidari, Zlatko Kopecki

**Affiliations:** ^1^ Future Industries Institute Adelaide University Adelaide South Australia Australia; ^2^ Women's and Children's Hospital Adelaide South Australia Australia; ^3^ College of Health, School of Pharmacy and Biomedical Sciences Adelaide University Adelaide South Australia Australia; ^4^ College of Medicine and Public Health Flinders University Adelaide South Australia Australia

**Keywords:** 16S rRNA sequencing, antimicrobial resistance, burn wounds, hydrogel, microbiome, paediatric, silver nanoparticles, *Staphylococcus aureus*

## Abstract

Burn injuries predispose paediatric patients to infection, and rising antimicrobial resistance (AMR) is reducing the effectiveness of conventional antibiotics. This underscores the need for novel antimicrobial dressings. This study characterised the microbiome and AMR profiles of paediatric burn wounds and assessed the antimicrobial efficacy of a silver nanoparticle (AgNP) hydrogel against clinical isolates. Twenty paediatric burn patients were recruited over a year, and 21 burn wounds were sampled. Three swabs were collected from each wound: one for full‐length 16S rRNA sequencing, one for species identification, and one for routine microbiology. Eleven wounds (from 10 patients) were clinically infected, and 10 were uninfected. Bacterial identification used standard culture methods and MALDI‐TOF. Antimicrobial susceptibility was determined using disk diffusion assays, and AgNP hydrogel efficacy was evaluated via agar‐well diffusion assay. Most patients were male (65%), under 5 years of age (55%), and had sustained scald burns (60%). Maximal burn depth significantly increased the likelihood of infection (*p* = 0.021) and subsequent systemic antibiotic use (*p* = 0.02). Clinically infected wounds exhibited significantly reduced alpha diversity, while beta diversity showed no distinct clustering by infection status. *Staphylococcus aureus* was enriched in infected wounds, whereas *S. epidermidis* predominated in uninfected wounds. Culture analyses revealed high penicillin resistance among *Staphylococci* and cefoxitin resistance in three coagulase‐negative strains. Five isolates demonstrated multidrug resistance. The AgNP hydrogel showed broad‐spectrum activity against all isolates, including multidrug‐resistant organisms. The AgNP hydrogel demonstrates antimicrobial activity against resistant burn wound pathogens. Large studies are required to support its clinical translation.

## Graphical abstract

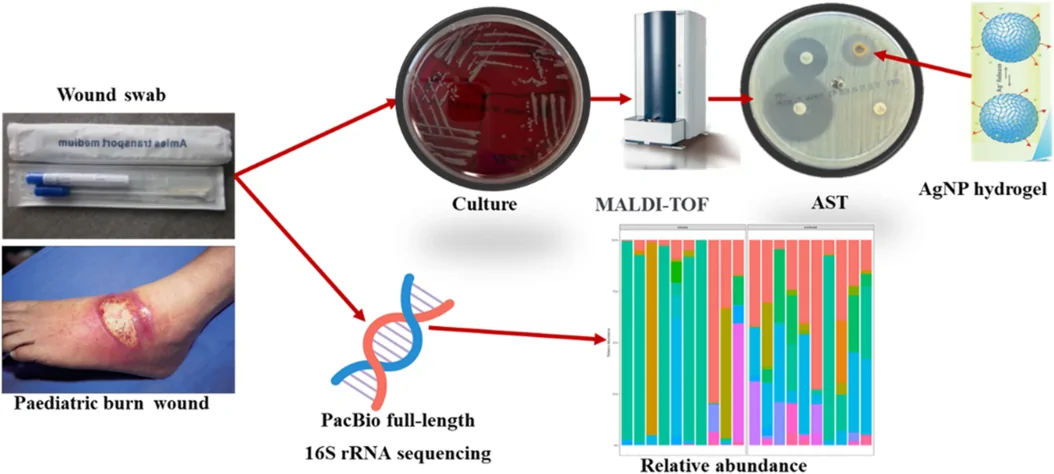

## 1 Introduction

Burn injuries are a major global health concern and remain a significant cause of morbidity (Jordan et al. 2022; Seah et al. 2019). Data from the 2019 Global Burden of Disease (GBD) study showed burns are the fifth most common cause of non‐fatal injury in children worldwide, affecting an estimated 2.65 million young lives under the age of 15 each year (Jordan et al. 2022; World Health Organization WHO Burns 2023). In Australia and New Zealand, 735 paediatric burn cases were treated across 16 specialist burn units in 2023 (Burns Registry of Australia and New Zealand Annual Report 2024). Scalds from hot liquids continue to be the leading cause of paediatric burns globally, particularly among toddlers under the age of 5 (Seah et al. 2019; Burns Registry of Australia and New Zealand Annual Report 2024; Hong et al. 2022; Chong et al. 2020).

While advances in wound care have dramatically improved survival rates for paediatric burns, the challenge is far from over with burn wound infections (BWI) remaining a major clinical challenge (Chong et al. 2020; Amsalu et al. 2025; Coban 2012). Infection significantly impacts morbidity by directly impairing wound healing (Vinaik et al. 2019), prolonging hospital stay with increased healthcare costs (Vinaik et al. 2019; Wanis et al. 2016), and severely escalates the risk of life‐threatening sepsis and death (Alp et al. 2012). Current effective management of BWI remains limited and challenged by growing bacterial resistance.

Children are more vulnerable to infections than adults because of several physiological differences, including their thinner skin, immature immune systems, larger surface area‐to‐body ratio, and a more dynamic, less resilient skin microbiome (Corcione et al. 2020). For example, children produce less sebum (oil), resulting in lower levels of beneficial lipophilic bacteria such as *Cutibacterium* on the skin (Zhu et al. 2019; Oh et al. 2012). These commensal microbes form a crucial first line of defence by supporting immunological, physical, and metabolic barrier functions that help inhibit pathogenic microorganisms (Zheng et al. 2020). Recent studies have shown that the skin microbiome undergoes significant changes following burn injury, including reduced bacterial diversity (Liu et al. 2018; Sanjar et al. 2020; Johnson et al. 2018) and dysbiosis—an imbalanced state where protective microbial diversity diminishes, and pathogenic microbes such as *Staphylococcus aureus, Acinetobacter baumannii* or *Pseudomonas aeruginosa* begin to dominate, often triggering clinical infection (Corcione et al. 2020; Horseman et al. 2023; Plichta et al. 2017; Park et al. 2017).

Currently, diagnosis of BWI relies largely on clinical signs, symptoms, and culture‐based microbiology (Ozhathil et al. 2025). Whilst culture‐based analysis is a surrogate for the actual wound microbiome, it often fails to capture the full complex picture of the wound's microbial ecosystem. Many species are difficult to cultivate under standard conditions, and this delays treatment programs (Paediatric Burns Service Guidelines 2023). Hence, clinicians are often forced to initiate broad‐spectrum antibiotics empirically with limited knowledge of the bacterial ecosystem and wound microbiome. This practice inadvertently contributes to the growing global crisis of antimicrobial resistance (AMR) by promoting the selection of resistant strains (Ho et al. 2025; Ajulo and Awosile 2024; Kalın et al. 2023). This includes methicillin‐resistant *S. aureus* (MRSA) and extended spectrum beta‐lactamase (ESBL)‐producing Gram‐negative bacteria, all of which are particularly concerning in paediatric burn patients (Amsalu et al. 2025).

Recent advances in high‐throughput sequencing have transformed our understanding of the wound microbiome, allowing researchers to identify not only different bacterial species, but also individual strains (Misic et al. 2014). Full‐length 16S rRNA offers significantly higher taxonomic resolution when compared to short‐read 16S rRNA sequencing (Ozhathil et al. 2025; Buetas et al. 2024). Although conventional culturing remains valuable, it is time‐consuming especially when analysing complex, polymicrobial samples, and sequencing alone cannot provide information on AMR profiles (Ozhathil et al. 2025). Despite these technological improvements, culture‐independent methods are still not routinely used in paediatric burn care. This underscores the urgent need for better infection monitoring, faster diagnostic tools, and more targeted antimicrobial treatments (Amsalu et al. 2025).

In this context, the development of novel antimicrobial dressings that prevent infection while modulating the wound microbiome, without fostering resistance, is a clinical and research priority (Gou et al. 2024). Among emerging technologies, silver nanoparticles (AgNPs)‐loaded smart hydrogels have shown promising antimicrobial properties (Gou et al. 2024). This is particularly due to its multifaceted mechanisms of action against a broad spectrum of pathogens, reduced toxicity, on‐demand silver ion release responsive to the wound microenvironment and wound healing potential (Haidari et al. 2024, 2020a, 2021a). In vitro and in vivo studies using bacterial laboratory strains have shown strong antimicrobial efficacy at substantially lower silver concentrations than commercial silver products (Haidari et al. 2024, 2021b). However, given the potential differences between clinical and laboratory‐adapted strains, it is essential to evaluate the performance of this AgNP‐loaded hydrogel against patient‐derived bacterial isolates from paediatric burn wounds. Currently, there is a limited number of studies assessing the performance of AgNP‐based products against clinical isolates, highlighting the need for further investigation to support their translational potential in clinical applications.

This study aims to characterise the wound microbiome in paediatric burn wounds, focusing on pathogen identification and their AMR profiles. Importantly, a comprehensive antimicrobial study was carried out to validate the susceptibility and sensitivity of the clinical isolates against our advanced ultrasmall (< 3 nm), on‐demand AgNP hydrogel mimicking a clinical infection scenario. These findings provide the descriptive microbial profile and resistance patterns in paediatric burn wounds, with implications for clinical management of burn patients in a specific healthcare setting in Australia and contribute to the development of more targeted, effective and safe alternative treatment strategies for the growing challenges of bacterial resistant infection in clinical settings. Further research with a larger cohort of paediatric burn patients across multiple study sites is required to validate these findings, strengthen the clinical relevance and inform changes to clinical practice.

## 2 Materials and Methods

### 2.1 Study Design and Clinical Data

A single‐centre, prospective cross‐sectional study was conducted from March 2024 to March 2025 at the Burns Unit of the Women's and Children's Hospital (WCH), Adelaide, Australia. Ethics approval for the collection of swabs from paediatric burn wounds was granted by the Women's and Children's Health Network Human Research Ethics committee, South Australia (2022/HRE00187) and the University of South Australia (HREC application number: 205564). All parents or guardians of patients provided their written informed consent prior to swab collection following the declaration of Helsinki ethical principles for medical research involving human subjects (Association 2013).

### 2.2 Study Population

#### 2.2.1 Inclusion Criteria

Paediatric burn patients aged 0–18 years with clinically infected and uninfected burn wounds managed at the WCH during the study period.

#### 2.2.2 Exclusion Criteria

Burn paediatric patients were excluded if their parents or guardians could not provide informed written consent. They were also excluded if they had received any systemic or topical antimicrobial treatment before the first wound swab, to avoid confounding effects on microbiome profiling and antimicrobial susceptibility testing. Information on subsequent antimicrobial use, both topical and systemic, administered after initial sampling as a part of routine clinical care was recorded to enable follow‐up of treatment outcome.

### 2.3 Recruitment Procedure

Paediatric patients with burn wounds were categorised as clinically infected and uninfected based on predefined clinical criteria, including signs of local infection (including erythema, swelling or tenderness, purulent discharge, offensive smell, increased pain, delayed healing or fever) and/or a positive microbiological culture result, as assessed by the treating clinician (WCHN 2023).

### 2.4 Specimen Collection and Transport

Twenty paediatric burn patients were recruited over a year, and 21 burn wounds were sampled. Three swabs were collected from each wound: one for full‐length 16S rRNA sequencing, one for species identification, and one for routine clinical care, for the local microbiology laboratory. Eleven wounds (from 10 patients) were clinically infected, and 10 were uninfected. Samples were obtained using the standard Levine collection technique (Song and Robinson 2021). Care was taken to avoid contamination from surrounding healthy skin flora. All swabs were placed immediately into Amies transport medium (Copan, TransSystem, Italy) and transported for same‐day microbiological and microbiome processing.

### 2.5 Genomic DNA Extraction for 16S rRNA Sequencing

One wound swab was used for Genomic DNA extraction using the PureLink microbiome DNA purification kit (Thermo Fisher Scientific, VIC, Australia) following the manufacturer's instructions. The absorbance of extracted DNA at 260 nm and 280 nm was measured using the Cytation5 imaging reader (BioTek Instruments, Winooski, Vermont, USA) and the Take3 Micro‐Volume plate and samples with an A260/A280 ratio of 1.8–1.9 were stored at −20°C prior to being sent to the Australian Genome Research Facility (AGRF) (Queensland, Australia) for full‐length16S rRNA sequencing. No measurable DNA was detected in the negative controls. Furthermore, genomic DNA integrity and purity were checked by AGRF using PCR gel electrophoresis before library preparation.

### 2.6 16S rRNA Sequencing Using Pacbio Kinnex Technology

To identify and quantify bacterial taxa present in paediatric burn wounds, full‐length 16S rRNA genes (V1‐V9 regions) were polymerase chain reaction (PCR) amplified using indexed primers: F27 (5'GCATC/barcode/AGRGTTYGATYMTGGCTCAG3') and R1492 (5'GCATC/barcode/RGYTACCTTGTTACGACTT3'). Amplicon libraries were generated using Kinnex full‐length RNA kits, and sequencing was performed using the SMRT Bell paired‐end sequencing chemistry on the PacBio platform by the AGRF.

### 2.7 Microbiome Bioinformatics and Statistical Analysis

To explore microbial communities in paediatric burn wounds, raw 16S rRNA sequencing data were processed using the Quantitative Insights into Microbial Ecology (QIIME2) pipeline (Bolyen et al. 2019). The data were denoised for inference of high‐quality amplicon sequencing variants (ASVs) using the Divisive Amplicon Denoising Algorithm (DADA2) plugin (Callahan et al. 2016). Taxonomic classification was performed using either VSERCH with a single reference database or a Naive Bayes classifier trained on three well‐established databases: SILVA rRNA database (v138), Genome Taxonomy Database (GTDB r207), and the NCBI RefSeq. 16S rRNA database supplemented by the Ribosomal Database Project (RDP) at 97% similarity (Johnson et al. 2019).

The feature‐table‐tax.biom and metadata (.tsv) files from QIIME2 output were imported into RStudio (version 4.4.1) using the phyloseq package (https://github.com/joey711/phyloseq) (McMurdie and Holmes 2013) for downstream analysis. Microbial diversity between clinically infected and uninfected burn wound samples was assessed using established ecological metrics including alpha and beta (β)‐ diversity analysis.

Alpha (α)‐ diversity, which describes the diversity within a single sample, reflects both microbial richness (number of taxa present) and evenness (distribution of taxa abundances). α‐ diversity was calculated using the Shannon diversity index (accounts for both richness and evenness), the Simpson index (dominance‐based diversity), and Observed species richness, which refers to the total number of distinct taxa (ASVs) detected in a sample (Shannon 1948). Differences in microbial diversity were statistically determined using the Wilcoxon rank‐sum exact test.

Beta (β)‐ diversity evaluates differences in microbial community composition between clinically infected and uninfected burn wounds. It measures how similar or dissimilar microbial communities are across clinically infected and uninfected wounds using a Bray–Curtis dissimilarity distance matrix (Bray and Curtis 1957) and visualised through principal coordinates analysis (PCoA). Statistical associations between infection status and distinct microbial community clustering patterns were evaluated using Permutational Multivariate Analysis of Variance (PERMANOVA) (Anderson 2017). Relative abundance of bacterial taxa was visualised through bar plots generated with the ggplot2 package (Wickham 2016). To identify potential microbial biomarkers associated with protective effects on BWI, with quantifiable effect sizes, linear discriminant analysis (LDA) Effect Size (LEfSe) was performed (Segata et al. 2011). Differentially abundant species‐level taxa were identified using pairwise Wilcoxon tests (*α* = 0.05), with a log LDA score > 2.0 used as the threshold for significance.

### 2.8 Genomic Data Availability

The PacBio full‐length 16S rRNA sequencing data have been deposited in the NCBI database under the Bio Project ID: PRJNA1349002.

### 2.9 Microbiological Cultures

The second swab was plated on various culture media, including MacConkey agar, 5% Columbia horse blood agar, Columbia colistin nalidixic acid supplemented sheep blood agar, mannitol salt agar, and Enterococcus agar plate (Edwards Group, Sydney, Australia). The plates were then incubated at 37°C for 24 h, and plates without growth were further incubated for up to 48 h. Colonies were differentiated by colour, form, haemolysis, consistency and purified by subculturing on the same plate. Following colony purification, morphologically distinct colonies were identified to the species level using matrix‐assisted laser desorption/ionisation time‐of‐flight mass spectrometry (Bruker Daltonik GmbH, Bremen, Germany). Confirmed bacterial isolates were stored at −80°C in 25% glycerol‐supplemented Tryptone soya broth (TSB) (CM0129, Thermo Scientific, Australia) (Sapula et al. 2023).

### 2.10 Antimicrobial Susceptibility Testing (Standard Antibiotics)

#### 2.10.1 Disk Diffusion Assay

The confirmed clinical isolates were tested using Kirby‐Bauer disk diffusion method (Bauer et al. 1966) against a panel of eight antibiotics included cefazoline (30 µg), cefoxitin (30 µg), amoxicillin‐clavulanic acid (20/10 µg), ciprofloxacin (5 µg), gentamycin (10 µg), trimethoprim‐sulfamethoxazole (1.25/23.75 µg), vancomycin (30 µg) and mupirocin (200 µg). Briefly, a single colony of each bacterial strain was inoculated into 5 mL cation adjusted Muller Hinton broth (caMHB) (Becton, Dickinson and Company, MD, USA) and incubated overnight at 37°C. Following incubation, the culture was vortexed, diluted 1:10 and adjusted to ~1 × 10^8^ colony‐forming units per mL (CFU mL^−1^) (0.5 McFarland standard) based on an optical density (OD600) reading using spectrophotometer. Using a sterile cotton swab, the bacterial suspension was evenly spread onto the surface of either caMH agar or Muller Hinton fastidious (MH‐F) medium which includes, caMH agar supplemented with 5% defibrinated sheep blood or 5% defibrinated horse blood, plus 20 mg/L *β*‐nicotinamide adenine dinucleotide media chosen according to species‐specific growth requirements. All procedures followed the European committee on antimicrobial Susceptibility testing (EUCAST, 2020) disk diffusion recommendations (Testing ECoAS 2020). Cefoxitin disk diffusion was used to detect the methicillin‐resistance phenotype of *Staphylococcus* species. Zone of inhibition demonstrating < 22 mm and 27 mm in diameter for *S. aureus* and *Coagulase*‐negative *Staphylococci* (CoNS) against cefoxitin were interpreted as MRSA and Methicillin‐resistant‐*CoNS* (MR‐*CoNS*), respectively according to the EUCAST breakpoint, 2025 (https://www.eucast.org/clinical breakpoints). Staphylococcal isolates that exhibited no zone of inhibition against 200 μg mupirocin disc were classified as having high‐level mupirocin resistance (MUP^H^), while those displaying any measurable zone of inhibition were considered absence of MUP^H^ resistance (Dadashi et al. 2020). Isolates categorised as intermediate or resistant to at least three antimicrobial classes were designated MDR (Magiorakos et al. 2012). *E. coli* ATCC 25922, S. *aureus* ATCC 25923 and *S. aureus* ATCC 43300 (MRSA) were included as control strains. All assays were performed in duplicate.

### 2.11 Preparation and Antimicrobial Efficacy of AgNP Hydrogel Against Clinical Bacterial Isolates

#### 2.11.1 AgNP Hydrogel Preparation and Synthesis

The preparation of the Pnipam‐PAA hydrogel and AgNP synthesis has been described in our previous work (Haidari et al. 2024). Briefly, N‐isopropylacrylamide (Nipam) and acrylic acid (AAc) monomers were polymerised via free‐radical polymerisation. The resulting hydrogel was purified with deionized water and dried at 65°C. Ultrasmall AgNPs were synthesised by forming Ag(I)–thiolate complexes from AgNO_3_ and mercaptosuccinic acid, followed by reduction with NaBH_4_ to generate stable nanoparticles, then loaded into the hydrogel by passive equilibration, yielding the final Pnipam‐PAA‐AgNP formulation without further chemical modification. The final product was used for all biological testings.

#### 2.11.2 Agar Well Diffusion

To evaluate the antimicrobial activity of hydrogel‐based formulations, the agar well diffusion technique was employed, as it is more suitable for semi‐solid materials than the disk diffusion method, which is optimal for impregnated filter paper disks. Briefly, a bacterial lawn was prepared as described above for the disk diffusion assay. Wells (6 mm diameter) were aseptically punched using a sterile biopsy punch, and 100 µL of the AgNP hydrogel, equivalent to 13 µg/ml of Ag, was added to each well. The blank hydrogel (without AgNPs) served as the formulation control. A ciprofloxacin (5 µg) disk was included as the positive control, while phosphate‐buffered saline (PBS) served as the negative control. All plates were incubated at 37°C for 24 h. Antimicrobial activity was expressed as the mean diameter of the zone of inhibition (mm).

#### 2.11.3 Minimum Inhibitory Concentration (MIC)

The MICs of the AgNP hydrogel were determined by first leaching the hydrogel in caMHB, followed by testing using the broth dilution method according to the ISO 20776‐1:2019 standard (https://www.iso.org/obp/ui/iso:std:iso:20776:-1:ed-2:v2:en) (EUCAST reading guide for broth microdilution 2024). In brief, two‐fold serial dilutions of leachate were prepared in a 96‐well microtiter plate (Corning, Victoria, Australia). Ciprofloxacin was included in parallel as a positive control. A single colony of each selected bacterial strain grown on nutrient agar (CM0309 Oxoid, Australia) plates was inoculated into caMHB, and the bacterial suspension was adjusted to 1×10^6^ colony‐forming units per mL (CFU mL^−1^). After 18–24 h of incubation at 37°C, bacterial growth was assessed by measuring OD at 600 _nm_ using spectrophotometer plate reader. The MIC was defined as the lowest concentration of the hydrogel leachate that inhibited visible bacterial growth. *S. aureus* ATCC 25923 and *S. epidermidis* ATCC12228 were included in each experiment for quality control. All MIC assays were performed with technical and biological replicates.

### 2.12 Statistical Analysis

Clinical and in vitro microbiological data were entered and analysed using Statistical Package for Social Sciences (SPSS), v26.0 software (IBM SPSS statistics, Armonk, NY). Chi‐square test, Fisher's exact test, independent samples *t*‐test and the Mann–Whitney U test were applied, as appropriate, to assess factors associated with the development of burn wound infection. A *p‐*value ≤ 0.05 was considered statistically significant.

## 3 Results

### 3.1 Patient Characteristics and Burn Profiles

Over a 1‐year period, a total of 20 paediatric patients with burn injuries were included in the study. Most patients were males (65%, 13/20), and toddlers (55% (11/20) under 5 years old (Table 1). The most common cause of burns was scald (60%), primarily from hot water, followed by contact burns (30%) and flame burns (10%). Most burns sustained partial‐thickness (superficial to deep dermal) (95%, 19/20) and small ≤ 5% TBSA (90%, 18/20) affected, while one patient presented with a full‐thickness (third degree) burn affecting 2% TBSA. The lower limbs were the most frequently affected body parts, involved in 60% of the cases. Encouragingly, 75% (15/20) of patients received proper first aid, defined as the application of cool running water for 20 min. One patient (WCH3) was treated with a cold pack, while four cases referred from external health centres, lacked documentation regarding first aid administration. Two patients (WCH6 and WCH7) had documented pre‐existing medical conditions, including autism spectrum disorder and congenital heart disorder, respectively. Only one patient (WCH27) required paediatric intensive care unit (PICU) admission due to a larger (12%) superficial to deep dermal scald injury.

**Table 1:** Comparison of demographic and burn‐related clinical characteristics between clinically infected and uninfected paediatric burn patients (*N* = 20).

Characteristics	Clinically infected (*n* = 10)	Uninfected (*n* = 10)	Total (*n* = 20)	*p*‐value
Age interval (years)				
< 1	1 (10)	2 (20)	3 (15)	0.566
1–4	5 (50)	3 (30)	8 (40)	
5–9	1 (10)	3 (30)	4 (20)	
10–15	3 (30)	2 (20)	5 (25)	
Gender				
Male	5 (50)	8 (80)	13 (65)	0.175
Female	5 (50)	2 (20)	7 (35)	
Primary cause				
Scald	6 (60)	6 (60)	12 (60)	0.264
Contact	4 (40)	2 (20)	6 (30)	
Flame	0	2 (20)	2 (10)	
TBSA, median (IQR)%	1 (3)	4 (5)	2.5 (2)	0.057
Degree of burn				
Partial thickness	9 (90)	10 (100)	19 (95)	0.5
Full thickness	1 (10)	0	1 (5)	
Maximal depth recorded				
Superficial dermal	1 (10)	6 (60)	7 (35)	**0.021**
Mid‐dermal	5 (50)	0	5 (25)	
Deep dermal	3 (30)	4 (40)	7 (35)	
Full thickness	1 (10)	0	1 (5)	
LOS, median (IQR)%	7 (7.25)	4 (12.5)	5.5 (11)	0.344
Antibiotic used during/after swab collection				
Yes	9 (90)	3 (30)	12 (60)	**0.02**
No	1 (10)	7 (70)	8 (40)	
First aid				
Yes	3 (30)	2 (20)	5 (25)	0.606
No	7 (70)^a^	8 (80)	15 (75)	
Excisional debridement				
Yes	4 (40)^b^	4 (40)	8 (40)	1.000
No	6 (60)	6 (60)	14 (60)	

^a^ One of clinically infected patient had cold pack application,

^b^ three patients underwent skin graft and received topical antibiotic bactroban (mupirocin), TBSA: total body surface area burned, IQR: inter quantile range, LOS: length of stay in hospitals, p‐value is based on either Chi‐square test, Fisher's exact test, independent samples t‐test, Mann–Whitney U test.

### 3.2 Antibiotic Use and Wound Care

Twelve patients received systemic antibiotics, primarily for clinical suspicion of infection (nine patients) or prophylactically (three patients) during febrile episodes without confirmed infection (Table 1). Among those treated, 91.7% (11/12) received either intravenous (IV) cefazoline or oral cefalexin. One patient received a combination of IV flucloxacillin, clindamycin and vancomycin. Most patients received silver‐based topical dressing (Acticot in 90% of cases, 18/20). Excisional debridement was performed in 40% (8/20) of patients, with three of these cases (WCH1, WCH7, and WCH9) undergoing skin grafting and receiving topical antibiotic Bactroban (mupirocin) (Figure S1).

### 3.3 Risk Factors Associated With Clinically Suspected Wound Infection

At the time of initial swab collection, 10 out of 20 patients were clinically suspected of having BWI. The development of BWI was significantly associated with the maximal burn depth (*p* = 0.021) and the use of systemic antibiotics (*p* = 0.02) during or after swab collection. Notably, every patient with a mid‐dermal burn developed an infection, while superficial dermal burns were predominantly uninfected. Other variables including age group (*p* = 0.566), gender (*p* = 0.175), primary cause of burn (*p* = 0.264), TBSA (*p* = 0.057), length of hospital stay (LOS) (*p* = 0.344), First Aid application (*p* = 0.60), and excisional debridement (*p* = 1.00) did not show a statistically significant link in this cohort (Table 1).

To assess the progression of infection and wound‐healing outcomes, microbiological data from the local laboratory and clinical information on time to complete wound healing (in days), defined as the period from admission to full epithelization of the wound, were retrospectively extracted from the hospital electronic medical records. Burn paediatric patients with a TBSA greater than 5% developed laboratory‐confirmed infections and experienced delayed wound healing compared to those with less than 5% TBSA (*p* = 0.05) (Figure S2).

### 3.4 Culture Based Isolation and Identification of Bacteria From Clinically Infected and Uninfected Burn Wounds

A total of 21 bacterial strains were isolated from burn wound swabs collected from 20 paediatric patients, including both clinically infected (*n* = 10) and uninfected (*n* = 10) groups. *Coagulase‐negative staphylococci* (CoNS) species were the most frequently isolated organisms (*n* = 10) followed by *S. aureus* (*n* = 4). Other isolates included *Streptococcus mitis_oralis* (*n* = 2), *Enterococcus faecalis, Corynobactrum propinquum* and *Arthrobacter gandavensis* (each *n* = 1). Notably, the Gram‐negative organisms *Acinetobacter lactucae* and *Entrobacter harmaechie* were exclusively isolated from clinically infected patients. These results closely matched with those obtained from the local microbiology laboratory, further supports the reliability of our culture‐based findings (Table S1).

### 3.5 Downstream Alpha and Beta Diversity Analysis Based on 16S rRNA Gene Sequencing

**Figure 1:**
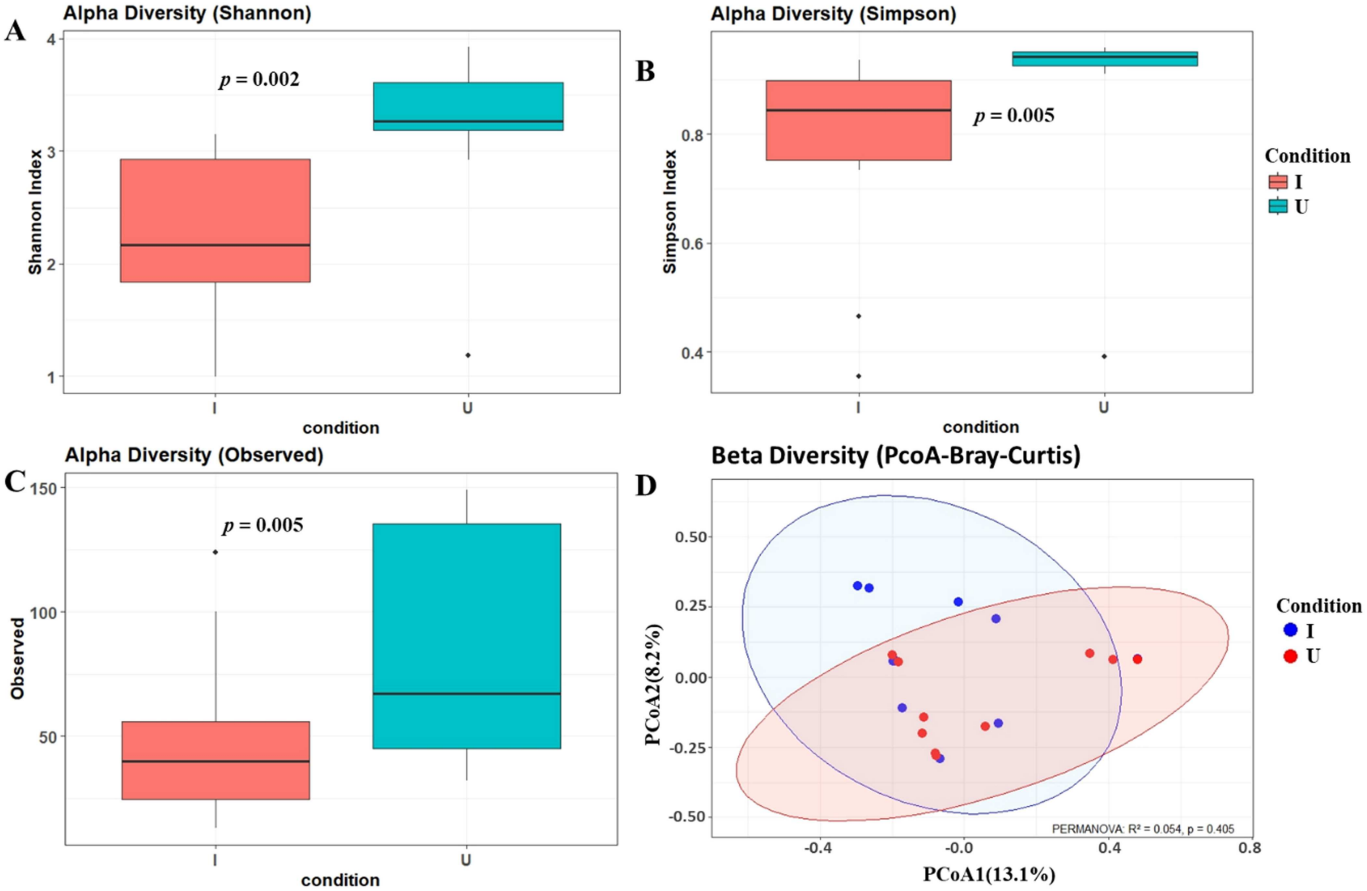
Alpha and beta diversity of microbial communities in clinically infected (I) versus uninfected (U) paediatric burn wounds based on full‐length 16S rRNA sequencing. (A) Shannon diversity index (B) Simpson diversity index (C) observed species richness, and (D) principal coordinates analysis (PCoA) plot based on Bray‐Curtis dissimilarity. Each dot represents an individual wound sample. Ellipses indicate 95% confidence intervals for clinically infected (light red) and uninfected (blue) groups, illustrating group‐level clustering.

Downstream alpha and beta diversity analyses were conducted to assess the composition and structure of the microbial communities in paediatric burn wounds using full‐length 16S rRNA gene sequencing. Alpha diversity across the three metrics (Shannon (*p* = 0.002), Simpson (*p* = 0.005) and observed species richness (*p* = 0.005)) revealed significantly reduced microbial diversity in clinically infected burn wounds compared to uninfected wounds (Figure 1A‐C). In contrast, beta‐diversity analysis using Bray‐Curtis dissimilarity revealed no statistically significant clustering between clinically infected and uninfected groups (PERMANOVA, R2 = 0.054, *p* = 0.408), indicating that only ~5.4% of microbial community was separated by infection status (Figure 1D).

Overall, both the alpha and beta diversity analyses suggest that while the community composition does not clearly separate by infection status, certain specific taxa may still differ in abundance.

**Figure 2:**
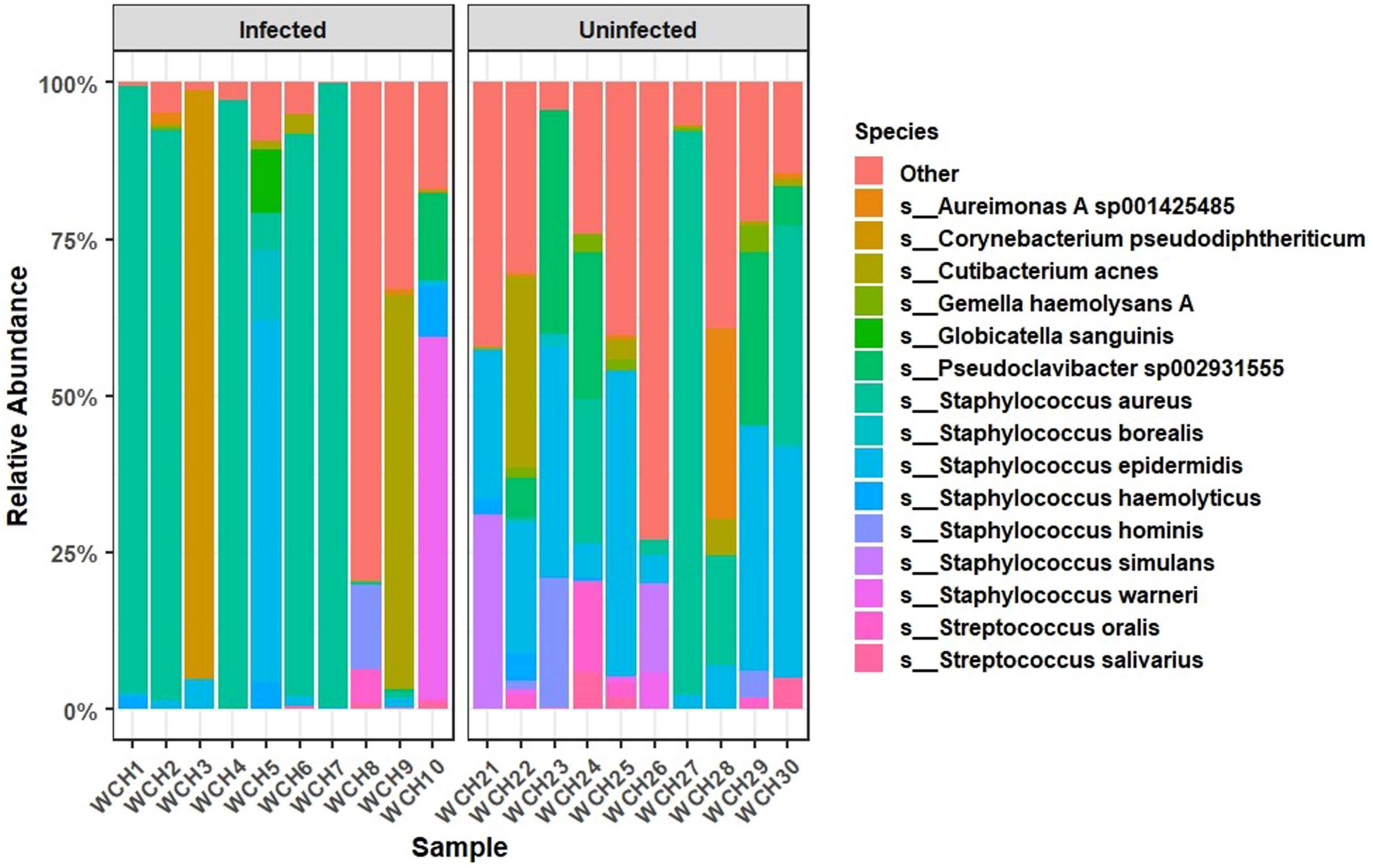
Stacked bar plot showing the relative abundances of the top 15 most abundant bacterial species detected in clinically infected (*n* = 10) and uninfected (n = 10) burn wound samples.

To further investigate this, we examined the relative abundance of individual species rather than large‐scale community shifts. The results from Figure 2 revealed that *S. aureus* was predominant in clinically infected wounds. In contrast, uninfected wounds showed higher microbial diversity, with no single species overwhelmingly dominating, instead showing significant presence of taxa typically considered non‐pathogenic skin commensals including *S. epidermidis*.

**Figure 3:**
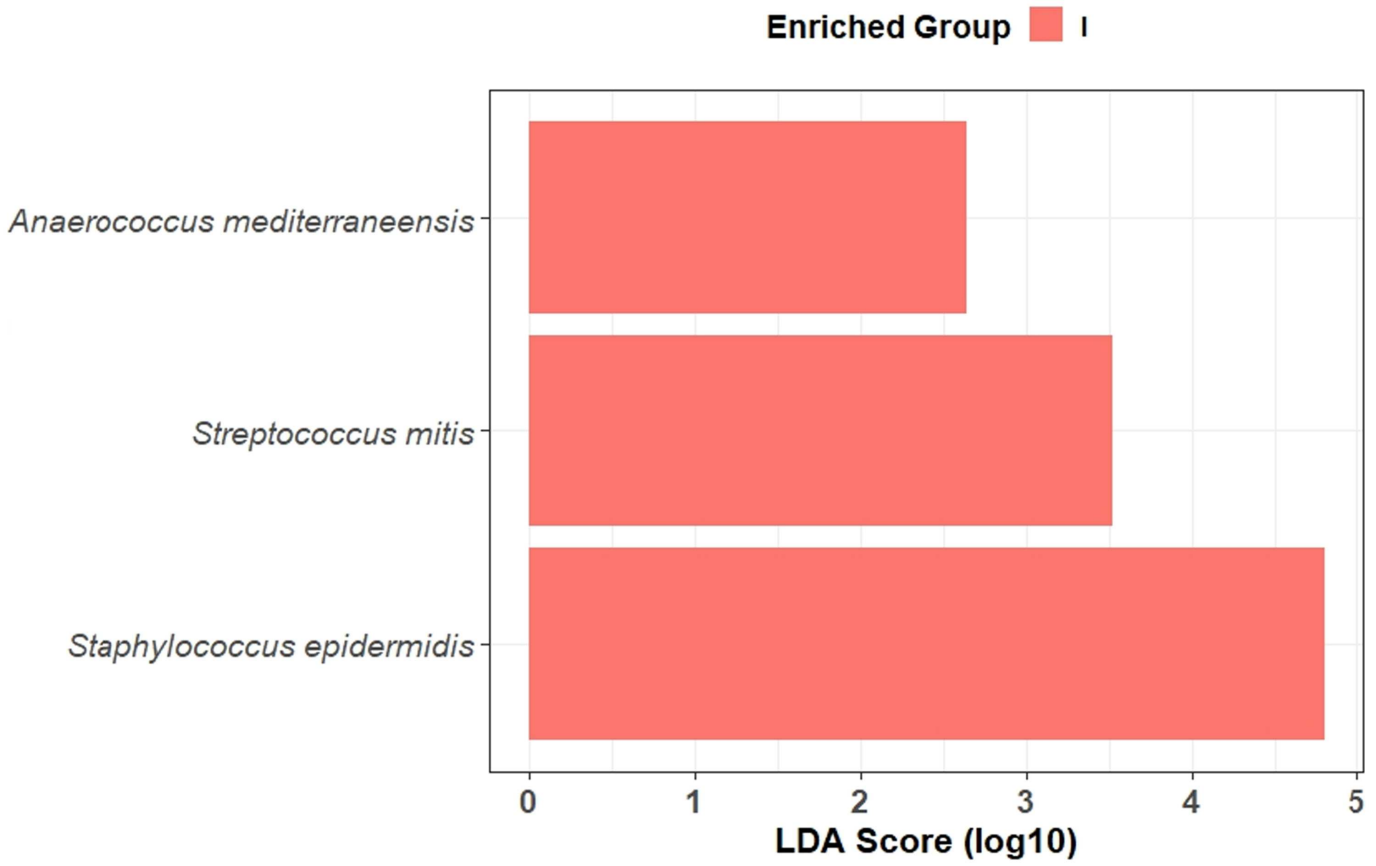
LEfSe analysis showing LDA scores of bacterial species significantly enriched in uninfected burn samples, using clinically infected samples as a baseline. The plot highlights the top species with LDA score > 2, suggesting differential abundance, of *S. epidermidis*, *S. mitis* and *A. mediterraneensis* enriched in the uninfected group.

To better understand the microbial differences between clinically infected and uninfected burn wounds, LEfSe analysis was employed on 16S rRNA sequencing data, using the clinically infected group as a baseline reference. This approach identified bacterial taxa showing differential abundance with relatively large effect sizes, which may suggest biologically relevant patterns between groups. The results from Figure 3 suggested that *S. epidermidis, Streptococcus mitis* and *Anaerococcus mediterraneensis* were more abundant in uninfected burn wounds compared to clinically infected wounds (LDA score > 2.6, *p* < 0.05). While these findings suggest potential differences in microbial composition, the findings should be interpreted with caution given the non‐significant beta‐diversity results. The observed enrichment of these taxa may reflect observed trends or potential shifts in microbial composition rather than definitive group separation. These species could contribute to maintaining microbial balance and limiting opportunistic pathogen colonisation; however, their functional role remains to be confirmed. Further large‐scale multi‐site studies are needed to validate these observations and explore the potential relevance of these findings for future design of novel targeted therapeutic strategies.

The dominance of the genus *Staphylococci* in the overall 16S rRNA sequenced samples (Figure S3) was supported by our culture‐based findings, which showed that *Staphylococci* species were the most frequently isolated organisms (66.7%, 14/21) (Table 2) consistent with other studies (Ke et al. 2023).

**Table 2:** Bacterial species isolated from burn wounds of paediatric patients.

Species	Number
Coagulase‐negative *Staphylococci* (CoNS)	10
*Staphylococcus aureus*	4
*Streptococcus mitis_oralis*	2
*Acinetobacter lactucae*	1
*Enterococcus faecalis*	1
*Corynobactrum propinquum*	1
*Entrobacter harmaechie*	1
*Arthrobacter gandavensis*	1
**Total**	**21**

*Note:* CoNS include Staphylococcus epidermidis, Staphylococcus caprae, Staphylococcus warneri, Staphylococcus haemolyticus, Staphylococcus hominis and Staphylococcus petrasii.

### 3.6 Antimicrobial Susceptibility Testing of Clinical Isolates From Burn Wounds

Of the 21 bacterial strains isolated from paediatric burn wounds, 85.7% (18/21) were resistant to at least one of the antibiotics tested. Most *Staphylococcus* species (85.7%;12/14) were resistant to penicillin. Among these, three CoNS demonstrated resistance to cefoxitin, indicating the presence of methicillin‐resistant CoNS strains. Notably, all tested *Staphylococcus* isolates were susceptible to mupirocin, a commonly prescribed antibiotic for wound infections (Table 3). The Gram‐negative isolates *A. lactucae* and *E. hormaechei* exhibited resistance to multiple β‐lactam agents, including cephalosporins and amoxicillin‐clavulanic acid, suggesting the presence of extended‐spectrum β‐lactamase (ESBL).

**Table 3:** Antimicrobial susceptibility profile of 21 bacterial strains isolated from paediatric burn wound swabs.

		Type of antimicrobials tested									
Sample ID	Bacterial species	AMC	CZO	CXI	CIP	GEN	SXT	MUP	PNG	CMN	MDR
WCH1	*Acinetobacter lactucae*	R	R	R	S	S	S	ND	ND	ND	NMDR
	*Staphylococcus aureus*	S	S	S	S	S	S	S	R	S	NMDR
	*Enterococcus faecalis* a	ND	ND	ND	S	R	R	ND	R	R	MDR
WCH3	*Corynebacterium propinquum*	S	S	S	S	S	R	ND	R	S	NMDR
	*Staphylococcus caprae*	S	S	S	S	R	S	S	R	R	MDR
WCH4	*Staphylococcus aureus*	S	S	S	R	R	S	S	R	R	MDR
WCH5	*Staphylococcus epidermidis*	S	S	S	R	S	S	S	R	S	NMDR
WCH6	*Streptococcus mitis_oralis*	S	S	S	S	R	R	S	S	S	NMDR
WCH7 Face	*Staphylococcus aureus*	S	S	S	S	R	S	S	R	S	NMDR
	*Enterobacter hormaechei*	R	R	R	S	S	S	ND	ND	ND	NMDR
WCH7 Shoulder	*Staphylococcus aureus*	S	S	S	S	R	S	S	R	S	NMDR
	*Staphylococcus epidermidis*	R	R	R	S	R	R	S	R	R	MDR
WCH9	*Arthrobacter gandavensis*	S	S	S	S	S	S	ND	R	S	NMDR
WCH10	*Staphylococcus warneri*	S	S	S	S	S	S	S	R	S	NMDR
	*Staphylococcus haemolyticus*	S	S	S	S	S	S	S	R	S	NMDR
	*Staphylococcus epidermidis*	R	R	R	S	S	S	S	R	S	NMDR
	*Staphylococcus hominis*	S	S	S	S	S	S	S	R	S	NMDR
WCH21	*Staphylococcus epidermidis*	S	S	S	S	S	S	S	S	S	S
WCH22	*Staphylococcus petrasii*	R	R	R	S	S	R	S	R	S	MDR
WCH25	*Streptococcus mitis_oralis*	S	S	S	S	S	S	ND	S	S	S
	*Staphylococcus epidermidis*	S	S	S	S	S	S	S	S	S	S

^a^ Vancomycin: sensitive, S: sensitive, NMDR: non‐multi‐drug resistant, MDR: multi‐drug resistant, AMC: amoxicillin‐clavulanic acid, CZO: cefazolin, CXI: cefoxitin, CIP: ciprofloxacin, GEN: gentamicin, SXT: trimethoprim‐sulfamethoxazole, MUP: mupirocin, PNG: benzylpenicillin, CMN: clindamycin, ND: not done

Among the 21 clinical isolates, 23.8% (5/21) were classified as multidrug‐resistant (MDR). Of these, three were CoNS, while the remaining two were *S. aureus* and *E. faecalis*. Interestingly, the MDR *S. aureus* isolate was susceptible to cefoxitin, and the MDR *E. faecalis* isolate was not resistant to vancomycin, the last resort antibiotic for Gram‐positive bacteria (Table 3).

### 3.7 Determining Antimicrobial Effect of AgNP Hydrogel on Clinical Isolates

The agar‐well inhibition assay was used to assess the antibacterial property of AgNP hydrogel and blank hydrogel against 13 clinical bacterial isolates from paediatric burn wound. As shown in Figure 3A,B, the blank hydrogel did not form an inhibition zone. However, AgNP hydrogel demonstrated a clear inhibition zone in all the organisms tested with consistent performance against different strains. The highest inhibition zone was seen in *S. hominis* (26 mm), a common microflora in the skin, whilst the smallest was observed for the MDR *E. faecalis* strain (13 mm) (Figure 3A,B).

**Figure 4:**
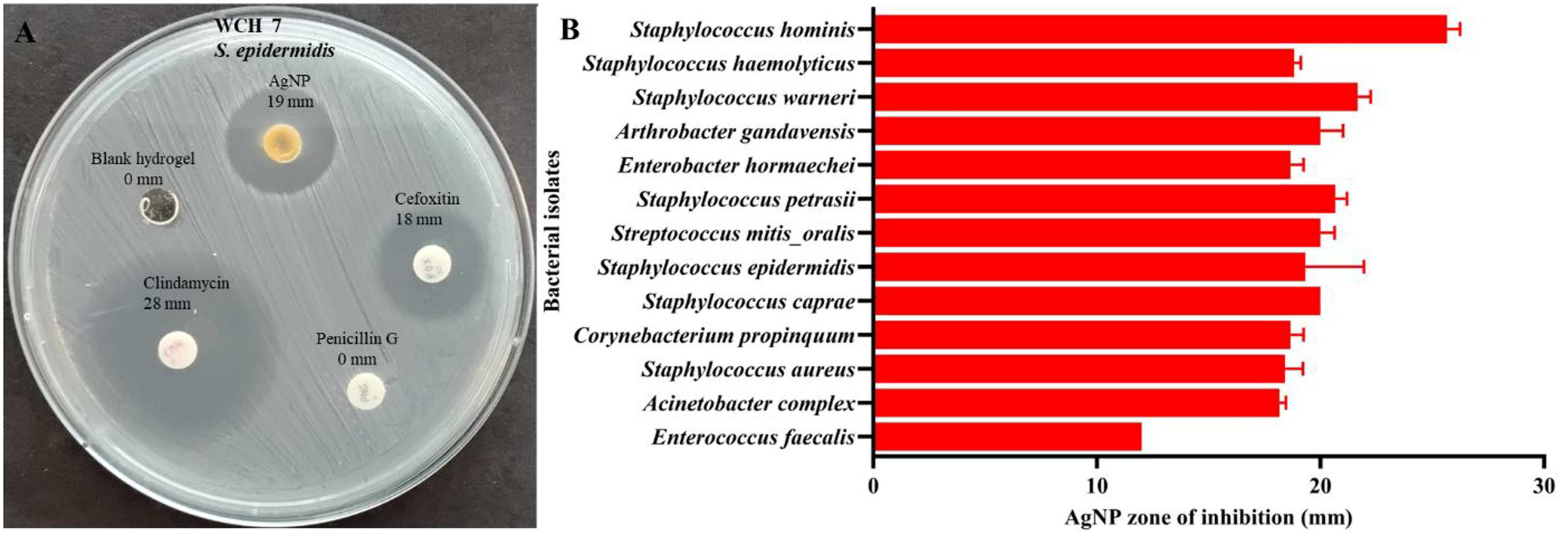
Antimicrobial activity of AgNP hydrogel against 21 bacterial isolates from paediatric burn wounds. (A) representative image of a zone of inhibition assay, (B) quantitative measurement of the zone of inhibition (in mm in diameter) for each of the 21 bacterial species.

Overall, AgNP hydrogel inhibits all isolates including cefoxitin resistant CoNS spp., which may harbour *mec* genes coding for *‘*methicillin’ resistance or high production of *Staphylococcal* β‐lactamase. The zone of inhibition was comparable to some of the tested antibiotics, demonstrating its strong potency against a broad spectrum of pathogens. Figure 4.

Furthermore, MIC testing was performed for selected clinical strains. The MIC values for *S. aureus* WCH1 and *S. epidermidis* WCH7 (5 µg/mL) were twofold higher than those of the corresponding laboratory reference strains (2.5 µg/mL) (Table S2). Moreover, the in vitro cytocompatibility of the AgNP hydrogel was evaluated using a resazurin and Live/Dead staining assays on primary human skin keratinocytes. The AgNP hydrogel demonstrated 96% viability, indicating good cytocompatibility in accordance with the ISO 10993‐5 standard, which defines materials with cell viability above 70% as non‐cytotoxic (Standardisation 2009) (Figure S4A–C). These findings suggest that the AgNP hydrogel may be safe for application to paediatric burn wounds.

## 4 Discussion

This study provides an in‐depth, species‐level characterisation of the microbial landscape in paediatric burns by combining conventional culture‐based identification with full‐length 16S rRNA sequencing. Additionally, it further assesses the clinical efficacy of a novel AgNP hydrogel dressing against the isolated pathogens, providing valuable insights that can inform multicentre, large‐scale studies supporting its clinical translation and therapeutic potential for managing paediatric wounds. This work is particularly significant given the escalating challenges associated with prescribed antibiotics for BWIs, which continued to show limited efficacy and increasing rates of resistance. The sharp rise in resistance observed over the last decade not only compromises treatment outcomes but also underscores the urgent need for alternative, effective, and targeted antimicrobial therapies to address the growing clinical burden of drug‐resistant infections in vulnerable paediatric populations.

Demographic and clinical characteristics of our cohort reflect national and global trends reported by the Australian and New Zealand Burn Association (ANZBA) (Riedlinger et al. 2015) and WHO Global Burn Registry (World Health Organisation WHO Burns 2023) and other epidemiological reports (Pointer and Tovell 2016). Most patients were male (65%) toddlers under 5 years of age (55%), and the predominant cause of burn injury was scalds from hot liquids (60%) (Jordan et al. 2022; Seah et al. 2019; Chong et al. 2020; Riedlinger et al. 2015; Nassar et al. 2023). The high proportion of partial thickness burns and low TBSA involvement is consistent with typical domestic accidents seen in this age group. This reflects known vulnerabilities in early childhood, where limited motor skills and curiosity often increase the risk of burn injury, which underscores the importance of interventions focusing on household hazards and parental education. Importantly, 75% of patients received standard first‐aid (cool running water for 20 min up to 3 h following injury) consistent with other Australian studies, where approximately 71.9% of paediatric burn patients were found to have received adequate first‐aid (Frear et al. 2021; Griffin et al. 2022). Studies have also shown that the provision of adequate First Aid is associated with improved outcomes in burns involving large body surface area (Harish et al. 2019).

Several studies to date have reported that burn depth and TBSA are independent predictors of BWI (Zhou et al. 2023; Posluszny JA et al. 2011). These findings were in line with our result that clinical infection status was significantly associated with burn depth. All patients with mid‐dermal burns developed infections, whereas superficial dermal burns were predominantly uninfected. This supports the hypothesis that deeper tissue injury compromises the skin barrier, promotes microbial invasion, and delays immune clearance (Penatzer et al. 2023). Unlike the previous study in the same hospital, no significant association was observed between infection status and TBSA (Amsalu et al. 2025), likely due to the small sample size. However, patients with larger > 5% TBSA exhibited late infection (Table S1) and prolonged wound healing times (Figure S2).

Both culture‐based and full‐length 16S rRNA gene sequencing consistently identified the Genus Staphylococcus, particularly *S. aureus* as dominant members of the burn wound microbiota. These findings are in line with previous surveillance studies, including a 13‐year retrospective analysis from the same hospital (Amsalu et al. 2025) and other reports (Park et al. 2017; DiMuzio et al. 2014). *S. aureus* is a widely recognised opportunistic pathogen frequently associated with delayed wound healing, biofilm formation, and the development of antibiotic‐resistant infections in burn patients (Haidari et al. 2020a).

Further downstream microbial diversity analysis of 16S rRNA sequencing revealed significantly reduced alpha diversity in clinically infected wounds compared to uninfected ones, supporting the concept of microbial dysbiosis as a hallmark of wound infection (Liu et al. 2018; Sanjar et al. 2020; Johnson et al. 2018). Although beta‐diversity (Bray‐Curtis) did not show significant clustering between groups, LEfSe analysis identified species enriched in uninfected wounds, including *S. epidermidis, S. mitis* and *A. mediterraneensis*. These commensals may contribute to colonisation resistance by outcompeting pathogens or modulating host immune response (Yang et al. 2024).

In this study, nearly 86% of Staphylococci isolates showed resistance to penicillin, consistent with the previous report (Amsalu et al. 2025). The persistently higher resistance to penicillin among Staphylococcus species may be attributed to the widespread use of cephalosporins in burn patients in which cefazolin/cephalexin was most often used. Additionally, three CoNS isolates were resistant to cefoxitin, indicating methicillin‐resistant phenotypes (MR‐CoNS). In contrast, no MRSA was identified in this study, differing from an earlier retrospective investigation in the same hospital, which reported a prevalence of 9.8% (Amsalu et al. 2025), as well as other Australian studies reporting MRSA rates among general burn patients (16%) (Issler‐Fisher et al. 2015) and 12.1% among bloodstream infection patients (Cleland et al. 2024). Gram‐negative isolates *A. lactucae* and *E. hormaechei* identified in this study demonstrated resistance to multiple β‐lactam antibiotics, including amoxicillin‐clavulanic acid, suggesting possible ESBL production. Overall, 23.4% of bacterial isolates exhibited MDR. The high prevalence of MDR bacteria in the paediatric burn unit may contribute to a vicious cycle of increasing antibiotic resistance, as clinicians are compelled to select empirical therapies targeting MDR organisms. Therefore, MR‐CoNS deserves more attention as one of the most common resident skin commensals which, following the disruption of the skin barrier and immune suppression caused by burns, can become opportunistic pathogens and are increasingly associated with BWI and bloodstream infections (Michels et al. 2021). A previous study in paediatric burn patients reported a significant increase in sepsis associated with AMR infections (Williams et al. 2009).

The novel AgNP hydrogel evaluated in this study demonstrated broad‐spectrum antimicrobial activity, including against MDR strains and cefoxitin‐resistant isolates. This aligns with our previous findings, which showed strong efficacy of the AgNP hydrogel against a range of pathogens and biofilms, in both in vitro and in vivo models (Haidari et al. 2020a, 2021a). The formulation has been shown to effectively reduce infection, modulate wound inflammation and support tissue repair.

Similar to the antimicrobial activity observed in our AgNP hydrogel, several commercially available silver‐based wound dressings and topical cream preparations, including aqueous silver sulfadiazine cream, AQUACEL Ag Extra, AQUACEL Ag+ Extra, ACTICOAT 7 and PROMOGRAN PRISMA matrix, have demonstrated bactericidal effects against both the Gram‐positive and Gram‐negative planktonic bacteria (Shrestha et al. 2024; Kostenko et al. 2010; Bourdillon et al. 2017). However, the continuous release of silver ions from these dressings has been associated with cytotoxic effects on host cells. Additionally, many currently available dressings provide different types of silver ions and deliver varying concentrations of silver ions to the wound bed in an uncontrolled manner, making it clinically difficult to standardise treatment protocols or clinical practice guidelines for the use of silver‐based treatments (Haidari et al. 2020b). While some progress has been made in last 6 years, the clinical challenges associated with silver‐based products remain (Shrestha et al. 2024). Lastly, AgNP wound dressings and topical preparations have been shown to provide reduced efficacy against established biofilms (Bourdillon et al. 2017). Notably, PROMOGRAN PRISMA matrix exhibited both antimicrobial and antibiofilm activity without adversely affecting host cell viability (Bourdillon et al. 2017). In contrast, the AgNP hydrogel developed in this study is designed to respond to local wound microenvironmental cues including temperature and pH, enabling on‐demand release of silver ions at optimal concentrations. This responsive behaviour allows for sustained, targeted antimicrobial activity while minimising potential cytotoxicity (Haidari et al. 2024, 2020a). Additionally, the multifaceted mechanisms of AgNP hydrogels, including disruption of the bacterial membranes, DNA damage, and oxidative stress, may contribute to the therapeutic effectiveness against resistant and biofilm‐forming organisms (Haidari et al. 2024, 2020a, 2021a). While these findings suggest potential advantages over conventional silver‐based dressings and currently available silver‐based topical preparations used clinically, direct comparative studies are required to confirm these benefits and further establish the clinical relevance of the developed AgNP hydrogel in paediatric burn care.

Despite the relatively small sample size (*n* = 20), which limited our ability to detect statistically significant associations for some clinical variables including TBSA, this study still provides meaningful insights into the relationship between burn depth, microbial dysbiosis, and infection in paediatric patients. However, the small cohort may reduce the overall statistical strength of the findings and may be influenced by the natural variability between individual patients, which is known to be particularly pronounced in burn wound microbiomes (Maitz et al. 2023). In addition, as this study was conducted within a single‐centre, there is a possibility of a cohort‐specific bias, and therefore the findings should be interpreted with some caution when considering broader clinical application. As this work provides a cross‐sectional snapshot, it does not fully capture how microbial communities change over time during the wound healing process. Further studies involving a large number of paediatric burn patients with different degrees of wound types across multiple centres will be important to validate these findings and strengthen clinical relevance. Longitudinal studies will help to better understand microbiome dynamics and their relationship with treatment outcomes, as well as further evaluate the safety and efficacy of the AgNP hydrogel in real‐world settings.

## 5 Conclusion

This study presents a combined phenotypic and molecular landscape of paediatric burn wounds, confirming a strong association between deeper burns, microbial dysbiosis, and infection with a high prevalence of MDR pathogens. In response to these clinical challenges, the novel AgNP hydrogel dressing demonstrated potent, broad‐spectrum antimicrobial activity against all clinical isolates tested in burn wounds, presenting a promising therapeutic approach. Overall, these findings underscore the need for longitudinal microbiome studies and support the integration of smart, non‐antibiotic strategies into antimicrobial stewardship practice and wound management protocols in this vulnerable population, to mitigate the escalating threat of AMR.

## Author Contributions

**Anteneh Amsalu:** conceptualisation; methodology; validation; visualisation; writing – review & editing; writing – original draft; software; formal analysis; data curation. **Anton Alvaro:** investigation; methodology; writing – review and editing. **Linda Quinn:** investigation; methodology; writing – review and editing. **Anna Antipov:** writing – review and editing. **Sylvia Sapula:** formal analysis; writing – review and editing; software. **Henrietta Venter:** writing – review and editing; formal analysis. **Bernard Carney:** validation; writing – review and editing; investigation. **Hanif Haidari:** methodology; writing – review and editing. **Zlatko Kopecki:** conceptualisation; funding acquisition; supervision; resources; project administration; writing – review and editing; writing – original draft; methodology.

## Ethics Statement

The authors have nothing to report.

## Conflicts of Interest

The authors declare no conflicts of interest.

## Supporting information


Supporting File

## Data Availability

The data that support the findings of this study are openly available in NCBI SRA at https://submit.ncbi.nlm.nih.gov/subs/.
