# Supplementary material for: Microbiome Profiling and Antimicrobial Efficacy of a Novel Silver‐Nanoparticle Hydrogel Dressing Against Clinical Isolates From Paediatric Burn Wounds
Source: Microbiologyopen. 2026 Aug 24;15(4):e70383. doi: 10.1002/mbo3.70383 (PMC13504237; doi:10.1002/mbo3.70383)
Supplement: Supplementary file 1 [file MBO3-15-e70383-s001.docx]

**Microbiome Profiling and Antimicrobial Efficacy of a Novel Silver-Nanoparticle Hydrogel Dressing against Clinical Isolates from Paediatric Burn Wounds**

*Anteneh Amsalu^1^, Anton Alvaro^2^, Linda Quinn^2^, Anna Antipov^1^, Sylvia Sapula^3^, Henrietta Venter^3^, Bernard Carney^2^, Hanif Haidari,^4^ Zlatko Kopecki^1^**

1. Future Industries Institute, University of South Australia, Mawson Lakes, SA 5095, Australia
2. Women’s and Children’s Hospital, Adelaide, South Australia, Australia
3. Clinical and Health Sciences, University of South Australia, City East, SA, 5000, Australia
4. College of Medicine and Public Health Flinders university, Bedford Park SA 5042

*Corresponding author

Dr Zlatko Kopecki,

zlatko.kopecki@unisa.edu.au

**Figure S1.** Distribution of antibiotics (red bar) and dressing types (black bar) administered to paediatric burn patients. Cefepime was administered to one patient who had been receiving cefazolin and subsequently tested culture positive.

**Wound healing (days)**

**TBSA (<5%)**

**TBSA (>5%)**

**Figure S2**. Box plot showing the length of wound healing time (in days) versus TBSA (%). Patients with larger burn areas (>5% TBSA) demonstrated longer and more variable healing times. Outliers are indicated by circles and asterisk.

**Figure S3.** Bar chart displaying distribution of most dominant Genus (15) and Species (20) identified in this study.

**Table S1**. Comparing culture result from the two-microbiology laboratory and follow-up culture result from the local microbiology.

| **Clinically Infected** | |  |  |  |  |  |  |  |  |
| --- | --- | --- | --- | --- | --- | --- | --- | --- | --- |
| **Lab.ID** | **Location of burn** | **Date of Burn** | **Date of swab** | **Date healed** | **Days for wound to heal** | **TBSA (%)** | **Depth** | **Culture from University of South Australia, Microbiology Laboratory** | **Culture result from Local Microbiology Laboratory** |
| WCH1 | Right ankle/foot | 14/03/2024 | 18/03/2024 | 10/04/2024 | 16.00 | 2 | Full thickness | *S. aureus, A. lactucae, E. faecalis* | *Staphylococcus aureus*, *Enterococcus faecalis*, Acinetobacter species, Coagulase negative staphylococci |
| WCH2 | Left leg | 24/03/2024 | 27/03/2024 | 5/04/2024 | 11.00 | 1 | Mid dermal | No growth | Skin flora |
| WCH3 | Face | 23/03/2024 | 27/03/2024 | 12/04/2024 | 19.00 | 1 | Superficial dermal | *C. propinguum, S. caprae* | *Cornybacterium propinquum*, Coagulase negative staphylococci |
| WCH4 | Bilateral Calves | 24/03/2024 | 30/03/2024 | 9/04/2024 | 15.00 | 1 | Mid dermal | *Staphylococcus aureus* | *Staphylococcus aureus* |
| WCH5 | Right calf | 29/03/2024 | 31/03/2024 | 12/04/2024 | 14.00 | 1 | Mid dermal | *Staphylococcus epidermidis* | Coagulase negative staphylococci |
| WCH6 | Abdomen and right arm | 5/04/2024 | 9/04/2024 | 23/04/2024 | 18.00 | 4 | Superficial and Mid dermal | *Staphylococcus aureus, Streptococcus mitis_oralis* | *Staphylococcus aureus* |
| WCH7 | Face, neck, shoulder | 21-Jun | 21/06/2024 | 24/10/2024 | 124.00 | 5 | Mid dermal | **Face** *Enterobacter hormaechei, S. aureus* **Shoulder** *S.epidermidis, S.aureus* | Neisseria sp, *Corynebacterium durum, Rothia aeria*, *Viridans streptococci,* **23/6/24** *Staphylococcus aureus* on blood cultures |
| WCH8 | Right foot | 1/07/2024 | 3/07/2024 | 30/07/2024 | 30.00 | 1 | Superficial to Deep dermal | No growth | No growth |
| WCH9 | Face, neck, chest | 13/07/2024 | 14/07/2024 | 14/08/2024 | 31.00 | 5 | Deep dermal | *Arthrobacter gandavensis* | **17/07/2024** *Seratia mercescens*, Coagulase negative Staphylococcic, *Actinomyces oris,* **25/07** Coagulase negative Staphylococci |
| WCH10 | Abdomen | 11/08/2024 | 16/08/2024 | 27/08/2024 | 16.00 | 1 | Deep dermal | *S. warneri, S. haemolyticus, S. epidermidis, S. hominis* | No growth |
| **Clinically uninfected** | | | | | | | | |  |
| WCH21 | Bilateral feet | 31/03/2024 | 31/03/2024 | 18/04/2024 | 19.00 | 2 | Deep dermal | *Staphylococcus epidermidis* | Coagulase negative Staphylococci |
| WCH22 | Bilateral leg | 3/04/2024 | 3/04/2024 | 23/04/2024 | 20.00 | 4 | superficial Dermal | *Staphylococcus petrasii* | No growth |
| WCH23 | Left thigh | 2/04/2024 | 3/04/2024 | 23/04/2024 | 23.00 | 4 | superficial Dermal | No growth | Coagulase negative Staphylococci |
| WCH24 | Abdomen, thigh | 9/04/2024 | 10/04/2024 | 23/04/2024 | 14.00 | 5 | Superficial dermal | No growth | *Staphylococcus aureus*, Viridans streptococci, Coagulase negative Staphylococci |
| WCH25 | Face, neck and chest | 19/04/2024 | 21/04/2024 | 8/05/2024 | 19.00 | 3 | Superficial to med dermal | *Staphylococcus epidermidis, Streptococcus mitis_oralis* | *Staphylococcus epidermidis* |
| WCH26 | Left foot | 20/04/2024 | 21/04/2024 | 14/05/2024 | 24.00 | 1 | Deep dermal | No growth | No growth |
| WCH27 | Face, chest, neck, arm | 9/05/2024 | 11/05/2024 | 26/06/2024 | 47.00 | 12 | Superficial, mid and deep Dermal | No growth | *Staphylococcus epidermidis*, *Viridans streptococci*, **14/05/24** *Staphylococcus aureus, Candida albicans*,**15/05/2024**, coagulase negative Staphylococci |
| WCH28 | Right thigh | 26/05/2024 | 31/05/2024 | 7/06/2024 | 11.00 | 2 | superficial Dermal | No growth | No growth |
| WCH29 | Chest, back | 27/05/2024 | 28/05/2024 | 19/06/2024 | 22.00 | 13 | Superficial dermal | No growth | *Staphylococcus aureus*, Acinetobacter spp., Coagulase negative Staphylococci, Lactococcus spp. |
| WCH30 | Left arm | 15/06/2024 | 18/06/2024 | 2/07/2024 | 17.00 | 4 | Superficial dermal | No growth | Coagulase negative Staphylococcus |

**Note:** The date listed in the Culture Result from Local Microbiology column represents follow-up laboratory results for patients who did not show clinical improvement under standard care. These results were retrospectively tracked to assess the progression of infection and microbiological outcomes.

**Table S2.** MIC of AgNP hydrogel as compared to ciprofloxacin.

| Bacterial species | *S. epidermidis* ATCC12228 | *S. aureus* ATCC 25923 | *S. aureus* (WCH1) | *S. epidermidis* (WCH7) |
| --- | --- | --- | --- | --- |
| CIP | 0.125 | 0.25 | 0.5 | 0.125 |
| AgNP | 2.5 | 2.5 | 5 | 5 |

MIC: minimum inhibitory concentration in µg/mL, CIP: ciprofloxacin, AgNP: silver nanoparticles

**Figure S4**.In vitro biocompatibility using resazurin assay against human keratinocyte cells. A) cell viability (%) after 24 h treatment B) representative images of results from resazurin assay for the respective treatments in the bar plot C) Live/Dead staining assay. Data are shown as mean values ± SD from three independent experiments with three technical replicates. Statistical analysis was performed using one-way ANOVA, ns=not significant (p > 0.05).
